# Complete genome sequence of *Salmonella enterica* subsp. *houtenae* from the oral cavity of a diseased blue-tongued lizard (*Tiliqua* sp.)

**DOI:** 10.1128/mra.00909-26

**Published:** 2026-09-23

**Authors:** Meng-Syun Li, Ter-Hsin Chen, Hue-Ying Chiou, Xin Ci Lim, Jong-Shian Liou, Chien-Hsun Huang, Ying-Chen Wu

**Affiliations:** 1 Graduate Institute of Veterinary Pathobiology, College of Veterinary Medicine, National Chung Hsing University 34916, Taichung, Taiwan; 2 Research Center for Animal Medicine, National Chung Hsing University 34916, Taichung, Taiwan; 3 Animal Disease Diagnostic Center, College of Veterinary Medicine, National Chung Hsing University 34916, Taichung, Taiwan; 4 Bioresource Collection and Research Center, Food Industry Research and Development Institute 63418, Hsinchu, Taiwan; University of Pittsburgh School of Medicine, Pittsburgh, Pennsylvania, USA

**Keywords:** *Salmonella*, *Tiliqua *sp., *in silico* serotyping, reptiles

## Abstract

A *Salmonella enterica* strain BT from the oral cavity of a diseased blue-tongued lizard was sequenced on the Oxford Nanopore platform. The assembled 4.8 Mbp genome consists of one circular chromosome and one circular plasmid (pBT). *In silico* serotyping suggested a rare serovar 40:z_4_,z_23_:- of the subspecies *houtenae* (IV).

## ANNOUNCEMENT

Over the past decades, reptiles have risen in popularity as pets worldwide (1). Given the close contact with their owners, serious concerns are raised on the zoonotic transmission of *Salmonella* spp., of which reptiles are known as reservoirs (2). In this study, we genome-sequenced a reptilian *Salmonella enterica* strain in order to determine its serovar. The Oxford Nanopore Technologies (ONT) platform was used, as it has been increasingly adopted for *Salmonella* surveillance (3, 4).

The blue-tongued lizard (*Tiliqua* sp.) was submitted for necropsy at the Animal Disease Diagnostic Center of National Chung Hsing University (NCHU) and diagnosed with enteritis and pneumonia. An oral swab collected for pathogen surveillance was inoculated onto sheep blood agar plates (Dr.plate Biotech), followed by 35°C aerobic incubation overnight. Colonies positive on *Salmonella* latex agglutination (Microgen) were sent to matrix-assisted laser desorption/ionization time-of-flight mass spectrometry (MALDI-TOF MS) analysis (Bruker) as previously described (5), and *Salmonella* sp. was identified with a score of around 2.0.

Genomic DNA was extracted from freshly prepared colonies using a Zymo Quick-DNA Fungal/Bacterial Miniprep Kit. The library was prepared using an ONT Rapid Barcoding Kit (SQK-RBK114.96), and sequencing was performed on the PromethION P24 platform equipped with an R10.4.1 flow cell, and the basecalling was made using the Dorado Super Accurate (SUP) basecalling model v4.3.0. The reads were filtered using Filtlong v0.2.1 (6) and assembled with Autocycler v0.5.2 (7). Contig analysis was performed using Bandage v0.8.1 (8). The final chromosome and plasmid sequences were rotated to start at *dnaA* and *RepB*, respectively, using dnaapler v1.3.0 (9). The genome quality was checked using CheckM2 (10). On 30 July 2026, the TYGS (11) was used to find the most closely related type strain for species and subspecies assignment, and PubMLST (12) was accessed to determine the sequence types. For annotation, the DFAST pipeline v1.4.0 (13) was capitalized on. SeqSero2 v1.3.1 (14), SISTR (15), and SSP (16) were used for *in silico* serotyping and ResFinder v4.7.2 (17) for prediction of antimicrobial resistance. Default parameters were used except where otherwise noted. Table 1 summarizes the outcomes of the analyses stated above, except for antibiotic resistance and serotyping.

**TABLE 1:** Summary of genome sequencing, assembly, annotation, and prediction results

Parameter	Strain BT
ONT sequencing	
No. of reads	170,814
Base count (bp)	759,278,925
Longest read (bp)	120,673
N50 (bp)	8,062
Assembly	
Size (bp)	4,802,745
Chromosome	4,682,036
Plasmid	120,709
Coverage (×)	
Chromosome	101
Plasmid	80
Completeness (%)	100
Contamination (%)	0.7
No. of coding DNA sequences (CDS)	4,537
No. of transfer RNA	83
No. of ribosomal RNA	22
No. of CRISPRs	1
GC content (%)	51.7
Coding ratio (%)	85.9
Most closely related type strain	*S. enterica* subsp. *houtenae* NCTC 10401^T*a*^
Seven-locus sequence type	ST-11805
Closest core genome sequence type	cgST-10819^*b*^

^*a*^ The digital DNA-DNA hybridization (dDDH) value (18) is 94.0% [91.5–95.8].

^*b*^ Of 2,750 loci, 2,439 (88.7%) were matched.

ResFinder predicted only tobramycin and amikacin resistance based on presence of *aac(6')-Iaa*; however, it has been discussed that this gene is rarely active at the transcriptional level (19).

**Fig 1:**
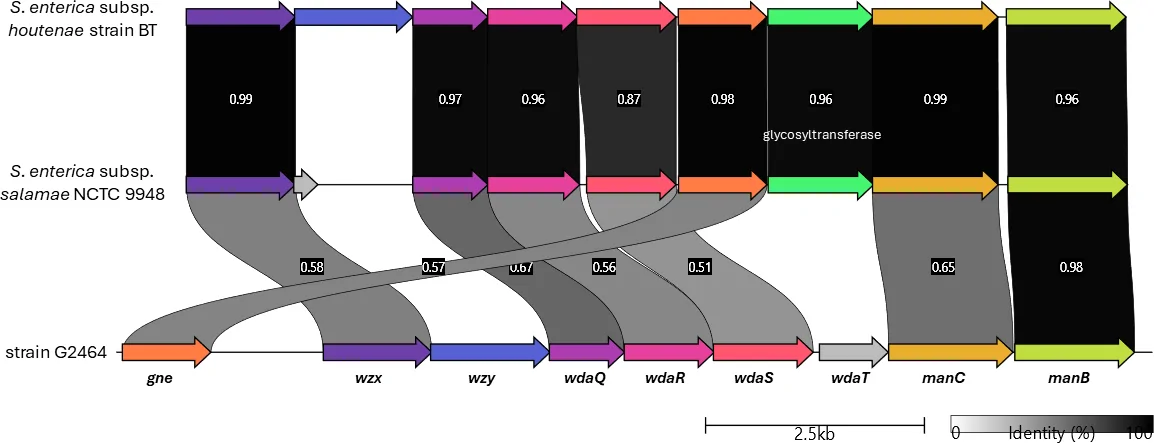
Comparative analysis of the O antigen gene cluster identified in strain BT with those of NCTC 9948 (serotype 1,40:z:1,5; LS483495.1) and strain G2464 (JX975331.1). The latter was included as it was used in a previous study on the genetic basis of *Salmonella* O antigens (22). The figure was plotted by clinker v0.0.32 (23).

SeqSero2 and SISTR detected no O antigen and predicted the antigenic profile as -:z_4_,z_23_:- but SSP gave 40:z_4_,z_23_:-. Interestingly, traditional serotyping (SSI Diagnostica Group) yielded a positive test on the polyvalent O and H antigen z_23_ antisera but was negative for z_4_. Regarding the conflicting O-antigen predictions, in light of the wet-lab evidence, we manually investigated the putative contributing gene cluster within the genome. The blastn analysis demonstrated that the identified genes share highest homology with known O40 antigen-bearing sequences (Fig. 1). And as z_4_,z_23_ was predicted consistently, we trusted this consensus and attributed the negative z_4_ test result to possible weak antigen expression or insufficient sensitivity of the antiserum. Accordingly, we concluded that this *S. enterica* strain BT belongs to IV 40:z_4_,z_23_:- according to the White-Kauffmann-Le Minor scheme (20, 21), a serovar rarely reported in the literature, even for reptiles.

## Data Availability

The complete genome sequence has been deposited in DNA Data Bank of Japan (DDBJ) under the accessions AP051740.1 (chromosome) and AP051741.1 (plasmid pBT), and the nanopore reads are publicly available via the DDBJ Sequence Read Archive under the accession DRR1078584. The BioProject accession is registered as PRJDB42779.
